# Emotional intelligence and innovative teaching behavior of pre-service music teachers: the chain mediating effects of psychological empowerment and career commitment

**DOI:** 10.3389/fpsyg.2025.1557806

**Published:** 2025-04-22

**Authors:** Xin Jiang, Yanli Tong

**Affiliations:** ^1^Department of Music, Universiti Malayai, Kuala Lumpur, Malaysia; ^2^Faculty of Language and Culture, Ningde Normal University, Ningde, China

**Keywords:** emotional intelligence, innovative teaching behavior, psychological empowerment, career commitment, pre-service music teachers, mediation effect

## Abstract

**Introduction:**

This study explores the chain mediating effects of psychological empowerment and career commitment in the relationship between emotional intelligence and innovative teaching behaviors among pre-service music teachers.

**Methods:**

A total of 458 pre-service music teachers (*M*_age_ = 22.56, *SD* = 1.97, 26.42% male, 73.58% female) participated in an empirical survey, employing the Emotional Intelligence Scale, Psychological Empowerment Scale, Career Commitment Scale, and Innovative Teaching Behavior Scale. Data analysis was conducted using structural equation modeling (SEM) with SPSS 26.0 and AMOS 24.0 to test the proposed mediation effects.

**Results:**

The results revealed significant positive relationships between emotional intelligence, psychological empowerment, career commitment, and innovative teaching behaviors. Mediation analysis demonstrated that emotional intelligence influences innovative teaching behaviors through the sequential mediation of psychological empowerment and career commitment.

**Discussion:**

These findings highlight the critical roles of psychological empowerment and career commitment in fostering innovative teaching behaviors. This study offers empirical evidence to enhance the innovative teaching capacities of pre-service music teachers and provides valuable implications for educational practice.

## 1 Introduction

Innovation is widely recognized as a key driver of sustainable development and organizational success (Fellnhofer, 2017; Nidumolu et al., 2009). Innovative behavior refers to the generation, development, and implementation of new ideas that improve job performance (Baskaran and Rajarathinam, 2018; Thurlings et al., 2015). In education, teacher innovation involves creating new ideas and significantly improving teaching methods (Li et al., 2024), enhancing both learning experiences and student engagement (Docherty et al., 2018). Teaching, beyond knowledge transmission, is a dynamic process of emotional exchange and regulation (Goran and Negoescu, 2015). Emotional interactions between teachers and students influence learning outcomes and educators’ professional development (Hargreaves, 1998; Jennings and Greenberg, 2009). Emotional Intelligence (EI) refers to the ability to recognize, understand, express, and regulate emotions in oneself and others, and to use this awareness to solve problems (Mayer et al., 2004; Salovey and Mayer, 1990). EI is conceptualized in two forms: trait EI, a self-perception of emotions, and ability EI, a cognitive capacity for managing emotions in real-world contexts (Mayer et al., 2008; Petrides et al., 2007). While trait EI and ability EI are distinct constructs (Gohm et al., 2005), this study adopts EI as a set of interrelated emotional processing abilities (Wong and Law, 2017). The role of EI in teaching has received increasing attention, as it influences teacher burnout, job satisfaction (Atmaca et al., 2020; Mérida-López and Extremera, 2017), and shapes student engagement and learning outcomes (Gumelar et al., 2024; Sowiyah and Zulaikha Fitriyanti, 2022). EI is a critical determinant of both individual and organizational effectiveness in education (Tripon, 2023).

Teacher education has traditionally focused on pedagogical knowledge and technical skills, often neglecting the emotional aspects of teaching (Harris and Sass, 2011). Although research has explored the emotional challenges faced by pre-service teachers (Hascher and Hagenauer, 2016; García-Martínez et al., 2021), the emotional dynamics in music education remain underexplored (Kirmizi and Sariçoban, 2020). Existing literature highlights the significant impact of EI on teachers’ effectiveness and well-being (Pilvera et al., 2024; Pyne, 2017), as well as on their professional engagement and job performance (Ismail et al., 2020; Mérida-López et al., 2023). While EI, Psychological Empowerment (PE), and Career Commitment (CC) have been explored in educational research (Hameli et al., 2023; Mérida-López and Extremera, 2020), the mechanisms through which EI influences innovative teaching behaviors in pre-service music teachers via PE and CC remain insufficiently explored.

Research on pre-service music educators has largely focused on professional identity and teaching self-efficacy (Bennett and Chong, 2018; Regier, 2021), with limited attention to their emotional experiences. Particularly within Chinese culture, the influence of EI and emotional experiences on innovative teaching behaviors is a critical gap. Understanding how emotional and psychological factors shape teaching innovation is essential for improving pre-service music teacher education. This study aims to fill this gap by examining how EI, PE, and CC interact to foster innovative teaching behaviors among pre-service music teachers.

### 1.1 Emotional intelligence and innovative teaching behavior of pre-service music teachers

Emotional intelligence is a form of social intelligence, involving the ability to monitor one’s own and others’ emotions and use this information to guide thoughts and actions (Mayer and Salovey, 1993; Salovey and Mayer, 1990). Teachers, as emotional professionals, must apply high EI to manage classroom dynamics and foster positive emotional connections with students (Bruney, 2012; Yin et al., 2019). For pre-service teachers, the internship phase bridges theory and practice, demanding not only pedagogical expertise but also the ability to manage teacher-student relationships and engage in effective emotional communication (Greve et al., 2020; Ramirez, 2020). During this phase, pre-service teachers use EI to meet teaching demands, build positive interactions, and strengthen their professional identities (Long et al., 2024). In addition, EI significantly influences teachers’ academic performance and professional development. For instance, Grehan et al. (2011) found a notable correlation between EI and academic outcomes (e.g., graduate GPA) as well as internship performance, underscoring the pivotal role of EI in enhancing both educational effectiveness and professional achievement.

Innovative behavior is multifaceted, involving both idea generation and the implementation of impactful innovations (Devloo et al., 2015). Teaching is inherently creative (Sawyer, 2011), and teachers’ innovative behaviors are key to enhancing student engagement and academic performance (Hosseini and Haghighi Shirazi, 2021; Khikmah, 2019). Studies have shown that high EI supports teachers’ work and promotes innovative teaching behaviors (TIB) (Mustafa et al., 2023; Pirkhaefi and Rafieyan, 2012; Su et al., 2022). The Conservation of Resources (COR) Theory suggests that individuals cope with stress by managing resources to achieve adaptive goals (Hobfoll, 1989). As a vital psychological resource, EI fosters innovative behaviors (Abraham, 1999; Görgens-Ekermans et al., 2015; Sapiee et al., 2024). The diverse demands of teaching itself act as resources that support the cultivation of innovative teaching behaviors (ITB) (Cao et al., 2020). Thus, EI plays a critical role in enabling pre-service music teachers to engage in innovative teaching behaviors during internships.

Based on the preceding discussion, this study proposes the following hypothesis:

*Hypothesis 1*: Emotional intelligence is positively associated with innovative teaching behaviors among pre-service music teachers.

### 1.2 The mediating role of psychological empowerment

Psychological empowerment (PE) is an intrinsic motivational construct consisting of four core dimensions: meaning, competence, autonomy, and impact (Spreitzer, 1995). Meaning reflects the perceived value of one’s work, competence pertains to the necessary skills for task completion, autonomy refers to the freedom in decision-making, and impact is the perceived effect of one’s contributions (Monje-Amor et al., 2021). These dimensions collectively shape individuals’ work behaviors, boosting motivation and fostering innovation. High EI has been shown to significantly enhance PE (Gong et al., 2020; Hameli et al., 2023). As a key internal driver, PE influences teachers’ professional behavior and supports the adoption of innovative practices (Zhang and Bartol, 2010; Zhu et al., 2019). From the perspective of Self-Determination Theory (SDT), fulfilling basic psychological needs—autonomy, competence, and relatedness—enhances intrinsic motivation (Deci and Ryan, 1985). Research shows that PE promotes active participation and autonomy, improving performance and driving innovation (Zhang and Bartol, 2010). By enhancing teachers’ perceptions of autonomy and self-efficacy, PE fosters greater engagement in teaching activities (Kõiv et al., 2019; Yorulmaz et al., 2018). Moreover, PE encourages teachers’ active involvement in innovative teaching strategies (Zhu et al., 2019). Despite growing recognition of PE as a key determinant of innovative behaviors (Singh and Sarkar, 2012), research on its antecedents, particularly in teaching, remains limited (Vermeulen et al., 2022).

Building on these insights, this study hypothesizes the following:

*Hypothesis 2*: Psychological empowerment mediates the relationship between emotional intelligence and innovative teaching behaviors among pre-service music teachers.

### 1.3 The mediating role of career commitment

Career commitment (CC) refers to an individual’s attachment and loyalty to their profession, reflecting a strong identification with and sustained dedication to one’s career (Blau, 1985; Huang et al., 2019; Jia et al., 2021). Research indicates that high levels of CC predict greater work engagement and overall well-being among teachers (Pourtousi and Ghanizadeh, 2020; Shu, 2022). For pre-service teachers, CC is also associated with lower dropout rates and reduced stress levels (Klassen and Chiu, 2011; Klassen et al., 2013). Moreover, CC plays a crucial role in shaping teachers’ professional behavior (Kim, 2012). Studies show that Emotional Intelligence (EI) positively influences both teachers’ work engagement and career commitment (Chesnut and Cullen, 2014; Mérida-López and Extremera, 2020; Sultana and Aldehayyat, 2018). According to the Conservation of Resources (COR) Theory, individuals manage stress by accumulating and protecting resources (Hobfoll, 1989; Hobfoll et al., 2018). When commitment wanes, effort and investment in work decrease (Wright and Hobfoll, 2004). In contrast, teachers with high CC are more likely to embrace innovative teaching practices to meet the evolving demands of education (Huang et al., 2019; Sena, 2020).

Based on the above theoretical and empirical insights, we hypothesize the following:

*Hypothesis 3*: Career commitment mediates the relationship between emotional intelligence and innovative teaching behaviors among pre-service music students.

### 1.4 The chain mediating role of psychological empowerment and career commitment

Previous studies have established a positive relationship between PE and CC among educators (Mabekoje et al., 2017; Winei et al., 2023). The satisfaction of basic psychological needs—autonomy, competence, and relatedness—has been identified as a key mediator in this relationship (Mabekoje et al., 2016). Empirical evidence suggests that educators with high levels of PE and CC exhibit greater professional engagement and commitment to their roles (Bogler and Somech, 2004). In the educational context, teachers’ EI fosters PE, which in turn promotes innovative behaviors (Khan et al., 2021; Shafait et al., 2021). Furthermore, research highlights that CC not only predicts teachers’ professional performance but also drives innovative work behaviors (Baharuddin et al., 2019). Teachers with higher CC are more likely to refine their teaching approaches and adopt innovative practices to improve classroom instruction (Asiyah et al., 2021; Thurlings et al., 2015).

Self-Determination Theory (SDT) clarifies that psychological empowerment promotes intrinsic motivation by satisfying basic psychological needs—autonomy, competence, and relatedness (Newman et al., 2017). These needs serve as mediators between PE and CC (Mabekoje et al., 2016). Satisfying these needs not only fosters the internalization of cultural values but also promotes a cohesive self-structure (Wilson et al., 2006), thereby enhancing intrinsic motivation and encouraging long-term career commitment (Gagné, 2014). Educators with higher levels of PE and CC demonstrate stronger professional engagement and commitment (Bogler and Somech, 2004). While previous research indicates that teacher empowerment and career commitment can independently mediate job satisfaction (Yao and Ma, 2024), multiple studies have confirmed their sequential mediation role. Specifically, psychological empowerment enhances teacher commitment, which in turn fosters innovative behavior and job performance (Qu et al., 2024; Xiaowei and Juan, 2019; Yao et al., 2024).

Based on these findings, the present study proposes a chain mediation model linking psychological empowerment and career commitment, with the following hypothesis:

*Hypothesis 4*: Psychological empowerment and career commitment act as chain mediators in the relationship between emotional intelligence and innovative teaching behaviors among pre-service music teachers.

**Figure 1:**
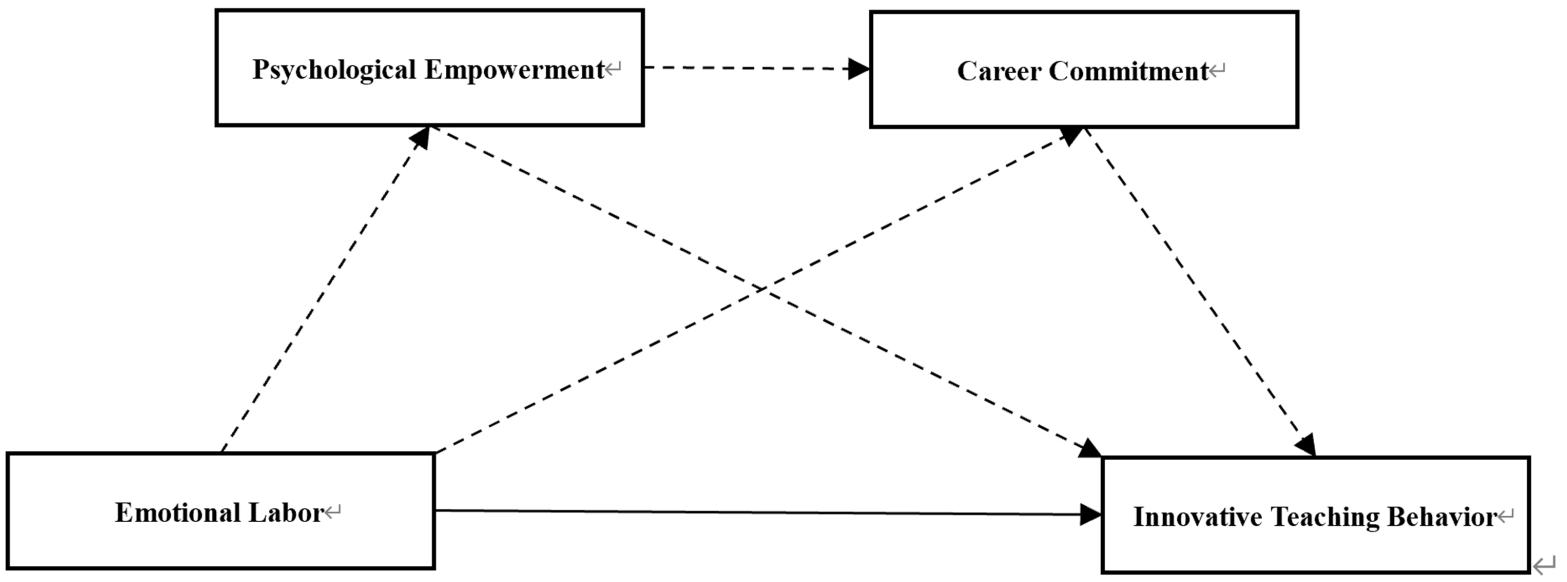
Hypothesized research model.

The proposed model illustrates the pathway through which emotional intelligence influences innovative teaching behaviors, with psychological empowerment and career commitment serving as sequential mediators, as depicted in Figure 1. This study examines the chain mediation effect of psychological empowerment and career commitment in teachers’ professional development, uncovering their potential impact on career growth. It provides new insights into both the theoretical and practical dimensions of education, enhancing the understanding of the interplay between psychological empowerment and career commitment.

## 2 Materials and methods

### 2.1 Participants

The participants were pre-service music teachers enrolled in music education programs at six universities in Eastern China. After excluding 58 incomplete responses from the initial 516 participants, the final sample consisted of 458 pre-service music teachers, yielding a response rate of 88.76%.

Table 1 summarizes the demographic characteristics of the participants. Among the 458 participants, 73.58% were female and 26.42% were male. The average age was 22.56 years (SD = 1.97), with the majority (61.79%) aged 20–22, followed by those aged 23–25 (26.64%), and those older than 25 (11.57%). The majority of participants (78.6%) held a bachelor’s degree, while 21.4% were graduate students. Regarding internship settings, 55.9% of participants interned in elementary schools, 32.1% in middle schools, and 12.01% in high schools. The typical internship lasted 8–12 weeks, with an average of 12.3 h per week spent directly interacting with students (SD = 3.1). Internships primarily involved core teaching activities, including classroom instruction, classroom management, and educational research.

**Table 1:** Demographic characteristics of the participants (*N* = 458).

Variable	Category	Frequency	Percentage (%)
Gender	Male	121	26.42
	Female	337	73.58
Age	20–22 years	283	61.79
	23–25 years	122	26.64
	Over 25 years	53	11.57
Education level	Undergraduate	360	78.60
	Graduate	98	21.40
School type	Primary	256	55.90
	Middle	147	32.10
	High	55	12.01

### 2.2 Measures

#### 2.2.1 Emotional intelligence (EI)

Emotional Intelligence was assessed using the Chinese version of the Emotional Intelligence Scale developed by Wong and Law (2002). The scale consists of 16 items across four dimensions: emotion regulation, self-emotion appraisal, others’ emotion appraisal, and use of emotion. Sample items include: “I can effectively control my emotions” (emotion regulation), “I always know whether I am happy” (self-emotion appraisal), “I am very sensitive to others’ feelings and emotions” (others’ emotion appraisal), and “I always tell myself that I am a competent person” (use of emotion). Participants responded on a 5-point Likert scale, ranging from 1 (“Strongly Disagree”) to 5 (“Strongly Agree”). The scale has been validated in previous studies on emotional intelligence among Chinese university students, demonstrating strong reliability and validity (Shengyao et al., 2024). In this study, the Cronbach’s alpha coefficient for the scale was 0.903, indicating excellent internal consistency.

#### 2.2.2 Psychological empowerment (PE)

Psychological empowerment (PE) was measured using the 12-item Psychological Empowerment Scale (PES), developed by Spreitzer (1995) and adapted for Chinese contexts by Li-Chaoping et al. (2006). The scale evaluates four dimensions: meaning (3 items), competence (3 items), self-determination (3 items), and impact (3 items). Participants rated each item on a 5-point Likert scale, where 1 represents “Strongly Disagree” and 5 represents “Strongly Agree.” Higher scores indicate greater levels of psychological empowerment. The scale has been previously validated in studies involving Chinese populations (Meng et al., 2015; Sun et al., 2022). In this study, the scale exhibited strong internal consistency, with a Cronbach’s alpha coefficient of 0.897.

#### 2.2.3 Career commitment (CC)

Career commitment was measured using the 12-item Career Commitment Scale developed by Carson and Bedeian (1994). This scale comprises three dimensions: career identity, career planning, and career dependence, with four items per dimension. Sample items include: Career Identity, “My job/career/field is personally meaningful to me”; Career Planning, “I have developed a plan for my career development in this job/career/field”; and Career Dependence, “The costs associated with my career sometimes seem excessively high,” which is reverse-scored. Participants rated each item on a 5-point Likert scale, with responses ranging from 1 (“Strongly Disagree”) to 5 (“Strongly Agree”). The participants in this study were pre-service teachers in the internship phase. Previous research demonstrates that this scale effectively measures career commitment among both pre-service teachers and internship students (Akinlolu and Chukwudi, 2019; Atikoh, 2022), and it has been successfully applied within the Chinese cultural context (Lin and Chen, 2020; Niu, 2010). The Cronbach’s alpha for this scale in the current study was 0.902, indicating good internal consistency. The original English version of the scale was translated into Chinese using translation software, and the translation was subsequently proofread and revised by a professional translator to ensure accuracy and appropriateness for the study context.

#### 2.2.4 Innovative teaching behavior (ITB)

Innovative teaching behavior was assessed using the Chinese version of the Teacher Innovative Work Behavior Scale developed by Zhang et al. (2012). The scale consists of three dimensions: innovative teaching ideas, innovative teaching actions, and innovative teaching outcomes. Example items include “I integrate innovative ideas into my teaching activities” for innovative teaching ideas, “I actively organize teaching activities to engage students in learning” for innovative teaching actions, and “Students in my classes achieve innovative outcomes, such as reports, products, processes, or activities” for innovative teaching outcomes. Participants rated each item on a 5-point Likert scale, where 1 denotes “Strongly Disagree” and 5 denotes “Strongly Agree.” Previous research has validated the use of this scale in studies examining innovative teaching behaviors among Chinese teachers (Li et al., 2017; Zhang et al., 2024). The Cronbach’s alpha for this scale was 0.924, indicating excellent internal consistency in this study.

### 2.3 Procedure and design

This study adopted a quantitative cross-sectional design to explore the relationships between EI, PE, CC, and ITB among pre-service music teachers in Eastern China. Data were collected through an online questionnaire distributed to music education students from six universities in Eastern China. The sample included senior undergraduate (fourth-year) and third-year graduate students, all of whom had completed their teaching internships prior to participation.

Data were collected via an online platform (e.g., “Wenjuanxing”), ensuring participants’ anonymity and confidentiality. The questionnaire was distributed and collected by the research team members, who ensured that participants received clear instructions about the study and how to complete the questionnaire. Participants were recruited using convenience sampling to ensure the accessibility and convenience of the sample. The survey was conducted in November 2024. Participants completed the questionnaire voluntarily, and the process took approximately 5–10 min per participant. The researchers explained the study’s purpose and provided detailed instructions for completing the questionnaire. Participation was voluntary, and participants could withdraw at any time without consequence.

Ethical approval was granted by the Ethics Committee of Ningde Normal University, and data collection began only after receiving this approval. Participants were informed of the study’s objectives, and assurances were provided that their responses would be used solely for academic research, with all responses anonymized to ensure confidentiality and privacy.

### 2.4 Data analysis

Data analysis was conducted using SPSS 26.0 and AMOS 24.0. The sample size for this study is deemed sufficient based on established guidelines. Tabachnick and Fidell (2013) recommend a minimum sample size of 300 for factor analysis, with the sample size being 5–10 times the number of predictor variables. Kline (2023) suggests that while 200 participants are adequate for Structural Equation Modeling (SEM), a larger sample is preferable. With 458 participants and four predictor variables, this study exceeds these recommendations, ensuring the reliability and validity of the statistical analyses.

To assess common method bias (CMB), Harman’s single-factor test was performed through exploratory factor analysis (EFA) on all measurement items (emotional intelligence, psychological empowerment, career commitment, and innovative teaching behavior). The analysis identified 13 factors with eigenvalues greater than 1, with the largest factor accounting for 33.12% of the total variance. This value is well below the 40% threshold, suggesting no significant common method bias. To further test for CMB, a common latent factor was introduced into the model, and confirmatory factor analysis (CFA) was performed. The results indicated that, after incorporating the common latent factor, neither the significance of the regression weights nor the model fit indices (TLI, CFI > 0.90, RMSEA < 0.05) changed substantially, providing additional evidence that no significant common method bias exists in this study (Podsakoff et al., 2003) (Table 2).

**Table 2:** Comparison of model fit indices.

Common indicators	Criteria	CFA model statistics	CFA with added common method factor statistics	∆
CMIN	–	1945.058	1620.060	–
DF	–	1,464	1,408	–
CMIN/DF	<3	1.239	1.151	–
RMSEA	<0.1	0.027	0.018	0.009
TLI	>0.90	0.966	0.985	0.019
CFI	>0.90	0.968	0.986	0.018

Subsequently, confirmatory factor analysis (CFA) was conducted to assess the model fit using AMOS 24.0. As shown in Table 3, the fit indices for both the individual latent variables and the overall model met the recommended thresholds ( $\frac{\chi 2}{df}\leq$ 3, RMSEA ≤ 0.08, CFI ≥ 0.90, TLI ≥ 0.90), suggesting a satisfactory fit for further analysis.

**Table 3:** Fit indices for confirmatory factor analysis.

Confirmatory factor analysis fit indices	*x* ^2^	*df*	*x*^2^/ *df*	RMSEA	CFI	TLI
EI	148.843	98	1.519	0.034	0.988	0.985
PE	77.930	48	1.624	0.037	0.989	0.985
CC	84.936	51	1.665	0.038	0.988	0.985
ITB	229.658	101	2.274	0.053	0.971	0.966
Overall model	1945.058	1,464	1.329	0.027	0.968	0.966

After performing confirmatory factor analysis (CFA), descriptive statistics and Pearson correlation analyses were conducted to examine the relationships among the study variables. The chain mediation effect was tested using the PROCESS macro (Model 6) for SPSS, with 5,000 bootstrap samples to generate 95% confidence intervals. The results confirmed the significance of the mediation effects. These statistical procedures enhance the robustness, validity, and precision of the findings, ensuring the reliability and accuracy of the analysis.

## 3 Results

### 3.1 Correlation analysis

Table 4 presents the Pearson correlation coefficients for the relationships among EI, PE, CC, and ITB. The analysis revealed significant positive correlations: EI was positively correlated with PE (*r* = 0.465, *p* < 0.01), CC (*r* = 0.611, *p* < 0.01), and ITB (*r* = 0.605, *p* < 0.01). Furthermore, PE was positively correlated with both CC (*r* = 0.524, *p* < 0.01) and ITB (*r* = 0.500, *p* < 0.01). Lastly, CC showed a positive correlation with ITB (*r* = 0.604, *p* < 0.01). These findings provide empirical support for the hypotheses tested in subsequent analyses.

**Table 4:** Results of the correlation analysis.

Variable	*M*	*SD*	Emotional intelligence	Psychological empowerment	Career commitment	Innovative teaching behavior
EI	3.625	0.710	1			
PE	3.807	0.713	0.465**	1		
CC	3.469	0.719	0.611**	0.524**	1	
ITB	3.457	0.720	0.605**	0.500**	0.604**	1

**p < 0.01.

### 3.2 Mediation effect testing

The chain mediation effect of EI on ITB, via PE and CC, was assessed using Model 6 of the SPSS PROCESS macro. A bootstrap procedure with 5,000 samples was applied to compute 95% confidence intervals for the estimated effects. As presented in Table 5, in the absence of mediator variables, EI significantly predicted ITB (*β* = 0.61, *t* = 16.24, 95% CI = [0.54, 0.69]), supporting Hypothesis 1. The chain mediation analysis further revealed that EI positively predicted PE (*β* = 0.47, *t* = 11.23, 95% CI = [0.39, 0.55]), which, in turn, positively predicted CC (*β* = 0.31, *t* = 7.77, 95% CI = [0.23, 0.39]). Additionally, EI (*β* = 0.33, *t* = 7.62, 95% CI = [0.25, 0.43]), PE (*β* = 0.19, *t* = 4.57, 95% CI = [0.11, 0.27]), and CC (*β* = 0.30, *t* = 6.62, 95% CI = [0.21, 0.39]) were all significant predictors of ITB. These results provide strong empirical support for Hypotheses 2 and 3.

**Table 5:** Results regarding the chain mediation model.

Result variables	Predictive variables	*R*	*R* ^2^	*F*	β	*t*	95% CI
ITB	EI	0.61	0.37	263.80***	0.61	16.24***	[0.54, 0.69]
PE	EI	0.47	0.22	126.13***	0.47	11.23***	[0.39, 0.55]
CC	EI	0.67	0.45	183.75***	0.47	11.90***	[0.40, 0.55]
	PE				0.31	7.77***	[0.23, 0.39]
ITB	EI	0.69	0.48	138.44***	0.33	7.62***	[0.25, 0.43]
	PE				0.19	4.57***	[0.11, 0.27]
	CC				0.30	6.62***	[0.21, 0.39]

Coefficients are standardized; bootstrap samples = 5,000; CI = confidence interval; ***p < 0.001.

**Figure 2:**
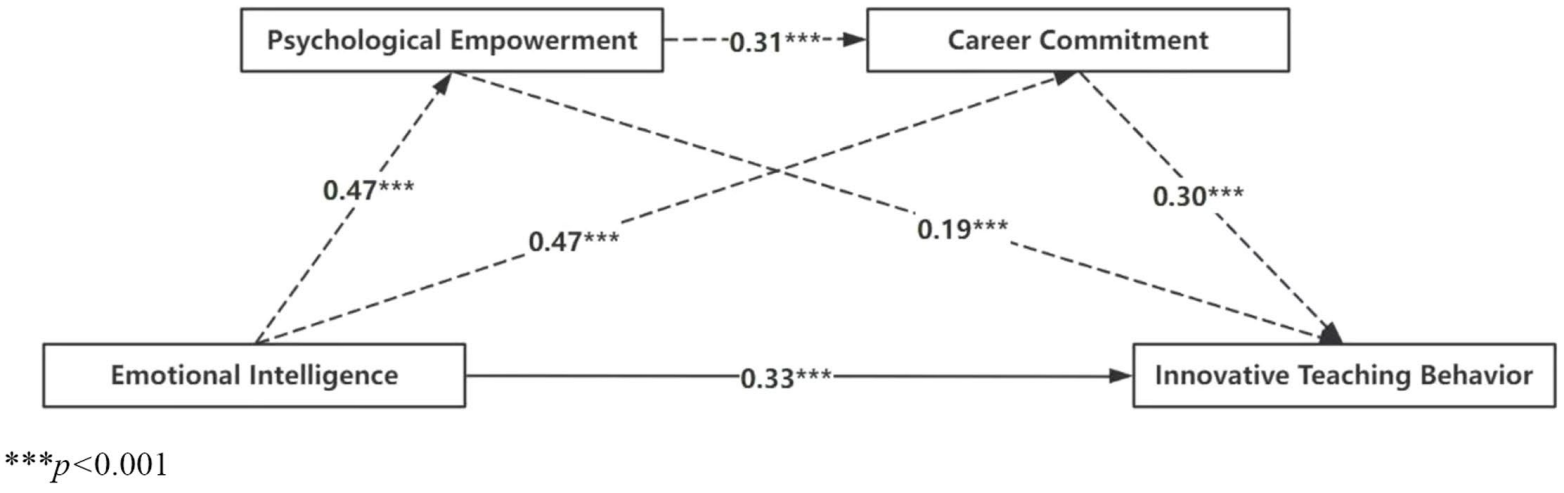
Results regarding the chain mediating effect.

The decomposition of the path effects is presented in Table 6. EI exerts significant indirect effects on ITB through both PE (Path 1: *effect* = 0.088, *SE* = 0.02, 95% CI = [0.04, 0.14], accounting for 14.36% of the total effect) and CC (Path 2: *effect* = 0.143, *SE* = 0.03, 95% CI = [0.09, 0.20], accounting for 23.33% of the total effect). Furthermore, the chain mediation effect via PE and CC (Path 3: *effect* = 0.044, *SE* = 0.01, 95% CI = [0.02, 0.07]) is also significant, representing 7.18% of the total effect. The total indirect effect was 0.275, accounting for 44.86% of the total effect, while the direct effect of EI on ITB was 0.338, representing 55.14% of the total effect. These findings provide strong empirical support for Hypothesis 4, demonstrating that PE and CC function as chain mediators in the relationship between EI and ITB. Figure 2 illustrates the path diagram of the chain mediation model, showcasing both the direct and indirect pathways through which EI influences ITB via PE and CC.

**Table 6:** Decomposition of the intermediary effect, direct effect, and total effect (*N* = 458).

Effect type	Paths	Effect	Boot	Boot	Boot	Effect percentage (%)
			*SE*	LLCI	ULCI	
Total effect	EI → ITB	0.613	0.04	0.54	0.69	
Direct effect	EI → ITB	0.338	0.04	0.25	0.43	55.14
Indirect effects	Total indirect effect	0.275	0.04	0.20	0.35	44.86
	Path 1: EI → PE → ITB	0.088	0.02	0.04	0.14	14.36
	Path 2: EI → CC → ITB	0.143	0.03	0.09	0.20	23.33
	Path 3: EI → PE → CC → ITB	0.044	0.01	0.02	0.07	7.18

Coefficients are standardized; bootstrap samples = 5,000; Boot LLCI, bootstrapping lower limit confidence interval; Boot ULCI, boot strapping upper limit confidence interval.

## 4 Discussion

This study investigates the impact of emotional intelligence (EI) on the innovative teaching behavior (ITB) of pre-service music teachers, with a specific focus on the chain mediation effects of psychological empowerment (PE) and career commitment (CC). The findings demonstrate that EI significantly enhances ITB, with PE and CC serving as critical mediators in this relationship. Furthermore, the chain mediation of PE and CC strengthens this association. These results contribute to a deeper understanding of the factors influencing innovative behavior in educational settings.

### 4.1 The relationship between emotional intelligence and innovative work behavior

The findings of this study provide strong evidence supporting the positive effect of EI on the innovative teaching behaviors of pre-service music teachers, thereby confirming Hypothesis 1. This result aligns with previous research, which highlights the essential role of emotional competence in music education, particularly given the unique emotional and artistic interactions inherent in this field (Girdzijauskas, 2015; Lasauskiene and Rauduvaite, 2015). EI improves teacher-student interactions (Friedman, 2014), enhances job satisfaction, and reduces burnout (D’Amico et al., 2020). Additionally, EI has been shown to foster innovative thinking and behaviors (Andrabi and Rainayee, 2020; Bonesso et al., 2020), and emotionally engaged teachers are more likely to design and implement innovative teaching strategies (Čábelková et al., 2022). Previous studies have shown that training programs focused on Social and Emotional Learning (SEL) significantly enhance pre-service teachers’ emotional intelligence (EI) without negatively impacting their academic performance in specific subjects (Gilar-Corbi et al., 2019; Özdemir Cihan and Dilekmen, 2024), further emphasizing the importance of fostering EI in pre-service teacher education. This study reinforces the positive role of EI in teaching. These findings are consistent with the Conservation of Resources (COR) theory (Hobfoll, 1989), which explains how individuals manage emotional regulation and respond to both positive and negative situations. As a critical resource, EI enables pre-service teachers to accumulate and optimize emotional resources, helping them navigate the complex demands of teaching and fostering innovative behaviors (Duman et al., 2014).

### 4.2 The mediating role of psychological empowerment

The results further demonstrate that PE significantly mediates the relationship between EI and ITB, thereby supporting Hypothesis 2. This finding extends previous research on the topic. PE, which reflects task autonomy, empowers individuals to control key aspects of their work, such as decision-making related to methods, procedures, pace, and effort (Spreitzer, 1995). By enhancing autonomy and competence, PE strengthens intrinsic motivation, thereby fostering greater engagement and performance (Yorulmaz et al., 2018). These results align with SDT, which posits that increasing autonomy and competence drives intrinsic motivation and promotes innovative behaviors (Bin Saeed et al., 2019; Ryan and Deci, 2000). In the context of pre-service teachers, positive emotions during internships contribute to the development of professional identity and teaching practices (Meyer, 2009), which in turn positively influence teaching methods and innovation (Ding and Hong, 2024). Previous studies have confirmed that EI positively influences PE, thereby enhancing its levels (Gong et al., 2020; Hameli et al., 2023). Moreover, PE serves as a crucial mechanism linking individual contributions to the outcomes of innovative projects (Malik et al., 2021). Teachers with higher levels of PE are better positioned to enhance their ITB (Zhu et al., 2019).

### 4.3 The mediating role of career commitment

The results of this study further reveal that CC mediates the relationship between EI and ITB, thereby supporting Hypothesis 3. According to the COR theory, individuals are motivated to acquire and protect valuable resources, such as organizational support. The accumulation of resources enhances workplace outcomes, whereas the loss of resources leads to stress and emotional depletion (Hobfoll et al., 2018; Westman et al., 2004). CC serves as a critical source of professional meaning and continuity, reinforcing intrinsic motivation and perseverance in one’s career (Chang, 1999). Moreover, the influence of EI on work-life balance and job satisfaction has been shown to strengthen career commitment (Marseno and Muafi, 2021). Individuals with higher career expectations and a strong sense of commitment are more likely to invest significant resources into their professional development (Goulet and Singh, 2002). For pre-service teachers, the internship phase plays a crucial role in fostering career commitment, as it supports the development of both professional identity and dedication to the teaching profession (Zhao and Zhang, 2017). Empirical studies consistently demonstrate a positive relationship between career commitment and innovative teaching behavior (Sena, 2020; Wahyuni et al., 2021). Therefore, EI not only contributes to enhancing pre-service teachers’ identification with the music education profession, but also facilitates their active engagement in innovative teaching behaviors, which ultimately supports their overall professional growth and development.

### 4.4 Chain mediating role of psychological empowerment and career commitment

The findings of this study further confirm that PE and CC jointly mediate the relationship between EI and ITB, providing strong support for Hypothesis 4. This chain mediation effect highlights the multifaceted role of EI in fostering innovation. Specifically, EI not only directly promotes innovation but also amplifies its impact by enhancing PE and strengthening CC. These results extend existing research, emphasizing the reciprocal relationship between PE and CC as key drivers of innovative behavior (Ambad and Bahron, 2012; Mabekoje et al., 2017).

Previous studies have shown that PE positively influences both CC and employee engagement (Mahmood and Sahar, 2017). The interplay between PE and CC has been recognized as a significant determinant of innovative work behavior (Huang et al., 2019; Yildiz et al., 2017). PE, in particular, serves as a crucial relationship between EI and effective teaching practices, enabling emotional regulation to translate into improved teaching outcomes (Hameli et al., 2023; Vrontis et al., 2020). This aligns with SDT, which underscores the importance of psychological needs for individual growth and well-being (Ryan, 1995; Van Den Broeck et al., 2008). By enhancing autonomy and competence, PE boosts intrinsic motivation, fostering greater engagement in innovative behaviors (Hameli and Ordun, 2022). Career commitment also acts as a motivational driver, encouraging teachers to invest in professional development and engage in more effective innovation (Hakimian et al., 2016; Wang and Hou, 2023). While research has mainly explored the positive impact of EI on PE and CC, limited attention has been given to whether career commitment can influence PE. Studies suggest that employee empowerment, through job security and growth opportunities, enhances satisfaction and organizational outcomes (Lau and May, 1998). Job security and rewards, in particular, foster psychological empowerment (Stander and Rothmann, 2010). Teachers with higher career commitment tend to take more initiative, improving job performance and strengthening emotional identity with their profession (Zhao and Zhang, 2017). These reverse mechanisms suggest that career commitment may influence psychological empowerment, indicating that future research should explore the bidirectional relationship between PE and CC to offer deeper insights into educational practice.

In sum, these results emphasize the critical mediating roles of psychological empowerment and career commitment, providing a deeper understanding of how emotional intelligence fosters teaching innovation. Specifically, EI not only enhances PE in pre-service music teachers but, through career commitment, further stimulates creativity and enthusiasm, fostering deeper engagement in innovative teaching practices. This study contributes a novel theoretical perspective to the field of music education, particularly regarding how EI enhances innovative teaching behaviors through PE and CC. It also offers valuable implications for educational practitioners, especially in fostering pre-service teachers’ EI, PE, and CC to better stimulate their innovative teaching potential.

## 5 Theoretical and practical implications

This study constructs a theoretical framework that integrates EI, PE, CC, and ITB, underpinned by Conservation of Resources Theory and Self-Determination Theory. The findings provide a deeper understanding of how EI influences pre-service music teachers’ innovative behaviors, with PE and CC acting as significant mediators. This extends existing research by highlighting the mechanisms through which EI fosters innovation, particularly in the context of music education. This study demonstrates how EI shapes pre-service music teachers’ ITB through two key mediators: PE and CC. By employing a serial mediation model, it offers a novel, systematic perspective on how EI drives ITB via multiple mediating mechanisms. This finding enriches the existing literature on the relationship between EI and innovation, particularly in the context of music education.

Moreover, the study underscores the importance of emotional regulation in the development of innovative teaching behaviors. By examining EI during pre-service music teachers’ internships, the research bridges emotional intelligence and practical innovation in music teaching, offering critical insights into how emotional management enhances creativity and performance in teaching. This study adopts a cross-sectional design, providing preliminary insights into the relationships among PE, CC, and ITB. While cross-sectional designs limit causal inference, they effectively highlight potential relationships, offering valuable perspectives for future longitudinal or experimental studies.

In terms of practical implications, the study recommends strategies to enhance the innovation of pre-service music teachers, emphasizing the key role of EI during the internship phase. Despite the limited presence of EI training in China (Ju et al., 2015), music education institutions should foster environments that support emotional regulation, helping pre-service teachers navigate emotional challenges in both teaching and artistic practice. The study underscores the importance of CC and PE in promoting ITB. For example, the National Teacher Training Program (NTTP) has successfully enhanced Chinese music teachers’ professional identity and self-efficacy (Yang, 2023). Strategically managing EI and fostering PE can support the professional and creative development of pre-service music teachers.

## 6 Limitations and future research directions

While this study provides valuable insights into the relationships among emotional intelligence (EI), psychological empowerment (PE), career commitment (CC), and innovative teaching behaviors (ITB), several limitations should be noted. First, the study employs a cross-sectional design based on self-reported data from a single time point, which limits the ability to draw causal inferences. Longitudinal studies or experimental designs that incorporate time-series analysis and manipulate independent variables while controlling for external factors would offer clearer insights into the causal relationships and their progression over time. Second, the sample was confined to pre-service music teachers from Eastern China, which may limit the generalizability of the findings to other geographical regions or cultural contexts. Replicating the study in diverse settings would enhance its external validity.

Although a formal power analysis was not conducted prior to data collection, the sample size (*N* = 458) exceeds the recommended thresholds for factor analysis and Structural Equation Modeling. Future studies may consider conducting a power analysis during the design phase to further confirm the adequacy of the sample size, ensuring optimal statistical power for detecting effects. Additionally, the study did not account for potential confounding factors such as professional competency and resilience (Wajdi et al., 2018; Pozo-Rico et al., 2023), which could influence the observed relationships. Future research could expand the model by incorporating additional variables, such as a moderated mediation model. Existing studies suggest that work engagement may mediate and self-efficacy may moderate the impact of EI on ITB (Su et al., 2022; Sun and Yuan, 2024). Moreover, psychological resilience has been shown to moderate the relationship between EI and individual performance (Kong et al., 2016). Although prior research confirms the validity of the Career Commitment Scale during transitional phases, career commitment may still fluctuate in such contexts. Future research should further explore the scale’s adaptability in capturing career instability.

Finally, the reliance on self-reported data introduces the possibility of social desirability bias. Future studies could adopt multi-method approaches, combining self-reports with objective data sources, such as behavioral observations, to enhance the robustness of the findings.

## 7 Conclusion

This study examines the impact of EI on ITB among pre-service music teachers, focusing on the mediating roles of PE and CC. The findings show that EI significantly enhances ITB, both directly and indirectly, by boosting PE and CC. Specifically, EI promotes innovation not only through direct influence but also by fostering psychological empowerment and career commitment. These results highlight the essential role of emotional intelligence in developing innovative capacity among pre-service music teachers. Future research should investigate how EI interacts with PE, CC, and other non-cognitive factors to shape ITB. Additionally, teacher training programs should incorporate strategies for managing emotional intelligence effectively, supporting pre-service music teachers’ engagement in innovative teaching practices.

## Data Availability

The raw data supporting the conclusions of this article will be made available by the authors, without undue reservation.

## References

[ref1] Abraham R. (1999). Emotional intelligence in organizations: a conceptualization. Genet. Soc. Gen. Psychol. Monogr. 125:209.

[ref2] Akinlolu A. D. Chukwudi A. R. (2019). Counselling self-efficacy and professional commitment: the mediating role of emotional intelligence and gender identification. Int. J. Sci. Res. Publicat. 9, 2250–3153. doi: 10.29322/IJSRP.9.03.2019.p8785

[ref3] Ambad S. N. A. Bahron A. (2012). Psychological empowerment: the influence on organizational commitment among employees in the construction sector. J. Glob. Bus. Manag. 8:73.

[ref4] Andrabi N. F. Rainayee R. A. (2020). Emotional intelligence and innovative work behaviour: a review. Int. J. Eng. Manag. Res. 10, 185–192. doi: 10.31033/ijemr.10.4.26

[ref5] Asiyah S. Wiyono B. B. Hidayah N. Supriyanto A. (2021). The effect of professional development, innovative work and work commitment on quality of teacher learning in elementary schools of Indonesia. Eurasian J. Educ. Res. 2021, 175–192. doi: 10.14689/ejer.2021.95.13

[ref6] Atikoh N. (2022). What did influence the students of the elementary preservice teachers’ commitment? A study on the effect of student’s self-leadership and self-concept. Al-Bidayah Jurnal Pendidikan Dasar Islam 14, 51–66. doi: 10.14421/albidayah.v14i1.767

[ref7] Atmaca Ç. Rızaoğlu F. Türkdoğan T. Yaylı D. (2020). An emotion-focused approach in predicting teacher burnout and job satisfaction. Teach. Teach. Educ. 90:103025. doi: 10.1016/j.tate.2020.103025

[ref8] Baharuddin M. F. Masrek M. N. Shuhidan S. M. (2019). Innovative work behaviour of school teachers: a conceptual framework. Int. J. Adv. Educ. 5, 213–221. doi: 10.18768/ijaedu.593851

[ref9] Baskaran K. Rajarathinam M. (2018). Innovative teaching practices in educational institutions (ITPEI). Int. J. Educ. Sci. 20, 72–76. doi: 10.1080/09751122.2017.1420599

[ref10] Bennett D. Chong E. K. M. (2018). Singaporean pre-service music teachers’ identities, motivations and career intentions. Int. J. Music. Educ. 36, 108–123. doi: 10.1177/0255761417703780

[ref11] Bin Saeed B. Afsar B. Shahjeha A. Imad Shah S. (2019). Does transformational leadership foster innovative work behavior? The roles of psychological empowerment, intrinsic motivation, and creative process engagement. Econ. Res. 32, 254–281. doi: 10.1080/1331677X.2018.1556108, PMID: 40101104

[ref12] Blau G. J. (1985). The measurement and prediction of career commitment. J. Occup. Psychol. 58, 277–288. doi: 10.1111/j.2044-8325.1985.tb00201.x

[ref13] Bogler R. Somech A. (2004). Influence of teacher empowerment on teachers’ organizational commitment, professional commitment and organizational citizenship behavior in schools. Teach. Teach. Educ. 20, 277–289. doi: 10.1016/j.tate.2004.02.003, PMID: 40177572

[ref14] Bonesso S. Cortellazzo L. Gerli F. (2020). Behavioral competencies for innovation: using emotional intelligence to foster innovation. Cham, Switzerland: Springer Nature. doi: 10.1007/978-3-030-40734-6

[ref15] Bruney G. (2012). The teacher-student relationship: the importance of developing trust and fostering emotional intelligence in the classroom. Master’s thesis, University of Toronto, Canada: University of Toronto Libraries. Available at: https://tspace.library.utoronto.ca/handle/1807/35096

[ref16] Čábelková I. Dvořák M. Smutka L. Strielkowski W. Volchik V. (2022). The predictive ability of emotional creativity in motivation for adaptive innovation among university professors under COVID-19 epidemic: an international study. Front. Psychol. 13:997213. doi: 10.3389/fpsyg.2022.997213, PMID: 36405122 PMC9669899

[ref17] Cao C. Shang L. Meng Q. (2020). Applying the job demands-resources model to exploring predictors of innovative teaching among university teachers. Teach. Teach. Educ. 89:103009. doi: 10.1016/j.tate.2019.103009, PMID: 40177572

[ref18] Carson K. D. Bedeian A. G. (1994). Career commitment: construction of a measure and examination of its psychometric properties. J. Vocat. Behav. 44, 237–262. doi: 10.1006/jvbe.1994.1017, PMID: 39885891

[ref19] Chang E. (1999). Career commitment as a complex moderator of organizational commitment and turnover intention. Hum. Relat. 52, 1257–1278. doi: 10.1023/A:1016908430206

[ref20] Chesnut S. R. Cullen T. A. (2014). Effects of self-efficacy, emotional intelligence, and perceptions of future work environment on preservice teacher commitment. Teach. Educ. 49, 116–132. doi: 10.1080/08878730.2014.887168

[ref21] D’Amico A. Geraci A. Tarantino C. (2020). The relationship between perceived emotional intelligence, work engagement, job satisfaction, and burnout in Italian school teachers: an exploratory study. Psihologijske Teme 29, 63–84. doi: 10.31820/pt.29.1.4

[ref22] Deci E. L. Ryan R. M. (1985). “Conceptualizations of intrinsic motivation and self-determination” in Intrinsic motivation and self-determination in human behavior. eds. Deci E. L. Ryan R. M. (New York, NY: Springer US), 11–40. doi: 10.1007/978-1-4899-2271-7_2

[ref23] Devloo T. Anseel F. De Beuckelaer A. Salanova M. (2015). Keep the fire burning: reciprocal gains of basic need satisfaction, intrinsic motivation, and innovative work behaviour. Eur. J. Work Organ. Psy. 24, 491–504. doi: 10.1080/1359432X.2014.931326, PMID: 40101104

[ref24] Ding L. Hong Z. (2024). On the relationship between pre-service teachers’ sense of self-efficacy and emotions in the integration of technology in their teacher developmental programs. Asia Pacific Educ. Res. 33, 869–878. doi: 10.1007/s40299-023-00758-6, PMID: 40177658

[ref25] Docherty A. Warkentin P. Borgen J. Garthe K. Fischer K. L. Najjar R. H. (2018). Enhancing student engagement: innovative strategies for intentional learning. J. Prof. Nurs. 34, 470–474. doi: 10.1016/j.profnurs.2018.05.001, PMID: 30527695

[ref26] Duman B. Göçen G. Yakar A. (2014). The examination of relationships between emotional intelligence levels and creativity levels of pre-service teachers in the teaching-learning process and environments. Pegem Eğitim Öğretim Dergisi 4, 45–74. doi: 10.14527/pegegog.2014.009

[ref27] Fellnhofer K. (2017). Drivers of innovation success in sustainable businesses. J. Clean. Prod. 167, 1534–1545. doi: 10.1016/j.jclepro.2017.08.197

[ref9001] Friedman S. (2014). Teacher emotional intelligence and the quality of their interactions with students (Doctoral dissertation). Rutgers University-Graduate School of Applied and Professional Psychology.

[ref28] Gagné M. (Ed.). (2014). The Oxford handbook of work engagement, motivation, and self-determination theory. New York, NY: Oxford University Press.

[ref29] García-Martínez I. Pérez-Navío E. Pérez-Ferra M. Quijano-López R. (2021). Relationship between emotional intelligence, educational achievement and academic stress of pre-service teachers. Behav. Sci. 11. doi: 10.3390/bs11070095, PMID: 34201438 PMC8301166

[ref30] Gilar-Corbi R. Pozo-Rico T. Pertegal-Felices M. L. Sanchez B. (2019). Emotional intelligence training intervention among trainee teachers: a quasi-experimental study. Psicologia: Reflexão e Crítica. 31:33. doi: 10.1186/s41155-018-0112-1 PMC6967020 32026146

[ref31] Girdzijauskas A. (2015). Seeking for value-based interpretation in musical instruction. Prob Music Pedagogy 14, 95–105.

[ref32] Gohm C. L. Corser G. C. Dalsky D. J. (2005). Emotional intelligence under stress: useful, unnecessary, or irrelevant? Personal. Individ. Differ. 39, 1017–1028. doi: 10.1016/j.paid.2005.03.018

[ref33] Gong Y. Wu Y. Huang P. Yan X. Luo Z. (2020). Psychological empowerment and work engagement as mediating roles between trait emotional intelligence and job satisfaction. Front. Psychol. 11:232. doi: 10.3389/fpsyg.2020.00232 32210866 PMC7067919

[ref34] Goran L. Negoescu G. (2015). Emotions at work. The management of emotions in the act of teaching. Procedia Soc. Behav. Sci. 180, 1605–1611. doi: 10.1016/j.sbspro.2015.02.314

[ref35] Görgens-Ekermans G. Delport M. Du Preez R. (2015). Developing emotional intelligence as a key psychological resource reservoir for sustained student success. SA J. Ind. Psychol. 41:13. doi: 10.4102/sajip.v41i1.1251

[ref36] Goulet L. R. Singh P. (2002). Career commitment: a reexamination and an extension. J. Vocat. Behav. 61, 73–91. doi: 10.1006/jvbe.2001.1844

[ref37] Grehan P. M. Flanagan R. Malgady R. G. (2011). Successful graduate students: the roles of personality traits and emotional intelligence. Psychol. Sch. 48, 317–331. doi: 10.1002/pits.20556, PMID: 40177840

[ref38] Greve S. Weber K. E. Brandes B. Maier J. (2020). Development of pre-service teachers’ teaching performance in physical education during a long-term internship. Ger. J. Exerc. Sport Res. 50, 343–353. doi: 10.1007/s12662-020-00651-0

[ref39] Gumelar W. S. Wulandari S. F. Lestari T. S. Ruswandi R. (2024). The correlation between teachers’ emotional intelligence and students’ learning engagement in EFL class. J. English Educ. Linguist. Stud. 11, 601–625. doi: 10.30762/jeels.v11i2.3377

[ref40] Hakimian F. Farid H. Ismail M. N. Nair P. K. (2016). Importance of commitment in encouraging employees’ innovative behaviour. Asia Pacific J. Bus. Administ. 8, 70–83. doi: 10.1108/APJBA-06-2015-0054, PMID: 35579975

[ref41] Hameli K. Ordun G. (2022). The mediating role of self-efficacy in the relationship between emotional intelligence and organizational commitment. Eur. J. Manag. Stud. 27, 75–97. doi: 10.1108/EJMS-05-2021-0033, PMID: 35579975

[ref42] Hameli K. Ukaj L. Çollaku L. (2023). The role of self-efficacy and psychological empowerment in explaining the relationship between emotional intelligence and work engagement. EuroMed J. Bus.. [Epubh ahead of preprint]. doi: 10.1108/EMJB-08-2023-0210

[ref43] Hargreaves A. (1998). The emotional politics of teaching and teacher development: with implications for educational leadership. Int. J. Leadersh. Educ. 1, 315–336. doi: 10.1080/1360312980010401

[ref44] Harris D. N. Sass T. R. (2011). Teacher training, teacher quality, and student achievement. J. Public Econ. 95, 798–812. doi: 10.1016/j.jpubeco.2010.11.009, PMID: 40177572

[ref45] Hascher T. Hagenauer G. (2016). Openness to theory and its importance for pre-service teachers’ self-efficacy, emotions, and classroom behaviour in the teaching practicum. Int. J. Educ. Res. 77, 15–25. doi: 10.1016/j.ijer.2016.02.003

[ref46] Hobfoll S. E. (1989). Conservation of resources: a new attempt at conceptualizing stress. Am. Psychol. 44, 513–524. doi: 10.1037/0003-066X.44.3.513, PMID: 2648906

[ref47] Hobfoll S. E. Halbesleben J. Neveu J.-P. Westman M. (2018). Conservation of resources in the organizational context: the reality of resources and their consequences. Annu. Rev. Organ. Psych. Organ. Behav. 5, 103–128. doi: 10.1146/annurev-orgpsych-032117-104640

[ref48] Hosseini S. Haghighi Shirazi Z. R. (2021). Towards teacher innovative work behavior: a conceptual model. Cogent Educ. 8:1869364. doi: 10.1080/2331186X.2020.1869364

[ref49] Huang W. Yuan C. Li M. (2019). Person–job fit and innovation behavior: roles of job involvement and career commitment. Front. Psychol. 10:1134. doi: 10.3389/fpsyg.2019.01134, PMID: 31156521 PMC6532537

[ref50] Ismail K. Nopiah Z. M. Rasul M. S. (2020). Emotional intelligence and work performance among vocational teachers. J. Tech. Educ. Train. 12, 106–117. doi: 10.30880/jtet.2020.12.03.011

[ref51] Jennings P. A. Greenberg M. T. (2009). The prosocial classroom: teacher social and emotional competence in relation to student and classroom outcomes. Rev. Educ. Res. 79, 491–525. doi: 10.3102/0034654308325693, PMID: 38293548

[ref52] Jia Y. Hou Z.-J. Wang D. (2021). Calling and career commitment among Chinese college students: career locus of control as a moderator. Int. J. Educ. Vocat. Guid. 21, 211–230. doi: 10.1007/s10775-020-09439-y, PMID: 40177658

[ref53] Ju C. Lan J. Li Y. Feng W. You X. (2015). The mediating role of workplace social support on the relationship between trait emotional intelligence and teacher burnout. Teach. Teach. Educ. 51, 58–67. doi: 10.1016/j.tate.2015.06.001

[ref54] Khan E. Khan M. M. Ahmed S. S. (2021). Transformational leadership, emotional intelligence, and innovative work behavior: the role of psychological empowerment. Global Manag. J. Acad. Corpor. Stud. 11, 63–76.

[ref55] Khikmah L. (2019). Teachers’ creativity in designing learning activities: sustaining students’ motivation. English Rev. J. English Educ. 7, 85–92. doi: 10.25134/erjee.v7i2.1639

[ref56] Kim Y. E. (2012). The effects of emotional labor on burnout and career commitment of childcare teachers. Korean J. Child. Educ. 8, 57–76.

[ref57] Kirmizi Ö. Sariçoban A. (2020). An investigation of the relation between pre-service EFL teachers’ emotions and their approaches to teaching. Dil Dilbilimi Çalışmaları Dergisi 16, 1968–1986. doi: 10.17263/jlls.851029

[ref58] Klassen R. M. Chiu M. M. (2011). The occupational commitment and intention to quit of practicing and pre-service teachers: influence of self-efficacy, job stress, and teaching context. Contemp. Educ. Psychol. 36, 114–129. doi: 10.1016/j.cedpsych.2011.01.002

[ref59] Klassen R. Wilson E. Siu A. F. Y. Hannok W. Wong M. W. Wongsri N.. (2013). Preservice teachers’ work stress, self-efficacy, and occupational commitment in four countries. Eur. J. Psychol. Educ. 28, 1289–1309. doi: 10.1007/s10212-012-0166-x

[ref60] Kline R. B. (2023). Principles and practice of structural equation modeling (5th ed.). New York, NY: Guilford Publications.

[ref61] Kõiv K. Liik K. Heidmets M. (2019). School leadership, teacher’s psychological empowerment and work-related outcomes. Int. J. Educ. Manag. 33, 1501–1514. doi: 10.1108/IJEM-08-2018-0232, PMID: 35579975

[ref62] Kong L. Liu Y. Li G. Fang Y. Kang X. Li P. (2016). Resilience moderates the relationship between emotional intelligence and clinical communication ability among Chinese practice nursing students: a structural equation model analysis. Nurse Educ. Today 46, 64–68. doi: 10.1016/j.nedt.2016.08.028, PMID: 27598795

[ref63] Lasauskiene J. Rauduvaite A. (2015). Expression of pre-service teachers’ emotional competency in their educational practice. Proc. Soc. Behav. Sci. 205, 103–109. doi: 10.1016/j.sbspro.2015.09.031

[ref64] Lau R. S. M. May B. E. (1998). A win-win paradigm for quality of work life and business performance. Hum. Resour. Dev. Q. 9, 211–226. doi: 10.1002/hrdq.3920090302

[ref65] Li M. Liu Y. Liu L. Wang Z. (2017). Proactive personality and innovative work behavior: the mediating effects of affective states and creative self-efficacy in teachers. Curr. Psychol. 36, 697–706. doi: 10.1007/s12144-016-9457-8

[ref66] Li K. Wijaya T. T. Chen X. Harahap M. S. (2024). Exploring the factors affecting elementary mathematics teachers’ innovative behavior: an integration of social cognitive theory. Sci. Rep. 14:1. doi: 10.1038/s41598-024-52604-4, PMID: 38267501 PMC10808225

[ref67] Li-Chaoping X. Shi-Kan Chen-Xuefeng. (2006). Psychological empowerment: measurement and its effect on employees’ work attitude in China. Acta Psychol. Sin. 38:99.

[ref68] Lin Y. Chen A. S. (2020). Experiencing career plateau on a committed career journey: a boundary condition of career stages. Pers. Rev. 50, 1797–1819. doi: 10.1108/PR-03-2020-0192

[ref69] Long J. Ying K. Luo Y. Chen X. (2024). Emotional intelligence development predicts novice teachers’ professional identity, teaching enthusiasm, and teacher-student relationships: the mediation of positive teacher emotions. Eur. J. Teach. Educ. Abingdon, Oxfordshire, UK: Routledge, Taylor & Francis Group. 47, 1–20. doi: 10.1080/02619768.2024.2414910, PMID: 40101104

[ref70] Mabekoje S. O. Azeez R. O. Bamgbose A. O. Okunuga O. O. (2017). The predictive and incremental validity of psychological empowerment dimensions on teachers’ career commitment beyond autonomy, competence and relatedness. Soc. Sci. 8, 197–209. doi: 10.1515/mjss-2017-0018

[ref71] Mabekoje S. O. Azeez O. Okunuga O. O. Bamgbose A. O. (2016). Does basic work needs satisfaction mediate between psychological empowerment and career commitment of teachers? AJIS. 5, 187–199. doi: 10.5901/ajis.2016.v5n3p187

[ref72] Mahmood A. Sahar A. (2017). Impact of psychological empowerment and perceived career support on employee work engagement with the mediating role of affective commitment. Pak. J. Commer. Soc. Sci. 11, 1084–1099.

[ref73] Malik M. Sarwar S. Orr S. (2021). Agile practices and performance: examining the role of psychological empowerment. Int. J. Proj. Manag. 39, 10–20. doi: 10.1016/j.ijproman.2020.09.002, PMID: 40177572

[ref74] Marseno W. A. Muafi M. (2021). The effects of work-life balance and emotional intelligence on organizational commitment mediated by work engagement. Int. J. Bus. Ecos. Strat. 3, 1–15. doi: 10.36096/ijbes.v3i2.257

[ref75] Mayer J. D. Salovey P. (1993). The intelligence of emotional intelligence. Intelligence 17, 433–442. doi: 10.1016/0160-2896(93)90010-3, PMID: 40174516

[ref76] Mayer J. D. Salovey P. Caruso D. R. (2004). TARGET ARTICLES: ‘emotional intelligence: theory, findings, and implications’. Psychol. Inq. 15, 197–215. doi: 10.1207/s15327965pli1503_02

[ref77] Mayer J. D. Salovey P. Caruso D. R. (2008). Emotional intelligence: new ability or eclectic traits? Am. Psychol. Washington, DC: American Psychological Association. 63, 503–517. doi: 10.1037/0003-066X.63.6.503 18793038

[ref78] Meng L. Liu Y. Liu H. Hu Y. Yang J. Liu J. (2015). Relationships among structural empowerment, psychological empowerment, intent to stay and burnout in nursing field in mainland China—based on a cross-sectional questionnaire research. Int. J. Nurs. Pract. 21, 303–312. doi: 10.1111/ijn.12279, PMID: 25521424

[ref79] Mérida-López S. Carvalho V. S. Chambel M. J. Extremera N. (2023). Emotional intelligence and teachers’ work engagement: the mediating and moderating role of perceived stress. J. Psychol. 157, 212–226. doi: 10.1080/00223980.2023.2169231 36808906

[ref80] Mérida-López S. Extremera N. (2017). Emotional intelligence and teacher burnout: a systematic review. Int. J. Educ. Res. 85, 121–130. doi: 10.1016/j.ijer.2017.07.006, PMID: 40177572

[ref81] Mérida-López S. Extremera N. (2020). When pre-service teachers’ lack of occupational commitment is not enough to explain intention to quit: emotional intelligence matters! Rev. Psicodidáctica 25, 52–58.

[ref82] Meyer D. K. (2009). “Entering the emotional practices of teaching” in Advances in teacher emotion research: the impact on teachers’ lives. eds. Schutz P. A. Zembylas M. (Springer US), 73–91.

[ref83] Monje-Amor A. Xanthopoulou D. Calvo N. Abeal Vázquez J. P. (2021). Structural empowerment, psychological empowerment, and work engagement: a cross-country study. Eur. Manag. J. 39, 779–789. doi: 10.1016/j.emj.2021.01.005, PMID: 40177572

[ref84] Mustafa H. G. Nageen S. Hussain S. Arif M. Siddique D. A. (2023). Emotional intelligence, work engagement, and creativity: a case study at university level in Pakistan. J. Positive Sch. Psychol. 7:6.

[ref85] Newman A. Schwarz G. Cooper B. Sendjaya S. (2017). How servant leadership influences organizational citizenship behavior: the roles of LMX, empowerment, and proactive personality. J. Bus. Ethics 145, 49–62. doi: 10.1007/s10551-015-2827-6, PMID: 40177658

[ref86] Nidumolu R. Prahalad C. K. Rangaswami M. R. (2009). Why sustainability is now the key driver of innovation. Harv. Bus. Rev. 87, 56–64.

[ref87] Niu H.-J. (2010). Investigating the effects of self-efficacy on foodservice industry employees’ career commitment. Int. J. Hosp. Manag. 29, 743–750. doi: 10.1016/j.ijhm.2010.03.006, PMID: 40177572

[ref88] Özdemir Cihan M. Dilekmen M. (2024). Emotional intelligence training for pre-service primary school teachers: a mixed methods research. Front. Psychol. 15:1326082. doi: 10.3389/fpsyg.2024.1326082, PMID: 38979067 PMC11228342

[ref89] Petrides K. V. Pita R. Kokkinaki F. (2007). The location of trait emotional intelligence in personality factor space. Br. J. Psychol. 98, 273–289. doi: 10.1348/000712606X120618, PMID: 17456273

[ref90] Pilvera S. C. Trinidad A. E. Sabud M. C. (2024). Building effective values educators: the role of emotional intelligence and instructional efficacy. Asian Res. J. Arts Soc. Sci. 22, 178–188. doi: 10.9734/arjass/2024/v22i12607, PMID: 40177820

[ref91] Pirkhaefi A. Rafieyan H. (2012). Investigation the relationship between emotional intelligence and mental health of primary school teachers with pupils’ creativity in Behshar city. Innovation and Creativity in Humanities. 4, 19–35. Tehran, Iran: Islamic Azad University, Science and Research Branch.

[ref92] Podsakoff P. M. MacKenzie S. B. Lee J.-Y. Podsakoff N. P. (2003). Common method biases in behavioral research: a critical review of the literature and recommended remedies. J. Appl. Psychol. 88, 879–903. doi: 10.1037/0021-9010.88.5.879, PMID: 14516251

[ref93] Pourtousi Z. Ghanizadeh A. (2020). Teachers’ motivation and its association with job commitment and work engagement. Psychol. Stud. 65, 455–466. doi: 10.1007/s12646-020-00571-x, PMID: 40177658

[ref94] Pozo-Rico T. Poveda R. Gutiérrez-Fresneda R. Castejón J.-L. Gilar-Corbi R. (2023). Revamping teacher training for challenging times: teachers’ well-being, resilience, emotional intelligence, and innovative methodologies as key teaching competencies. Psychol. Res. Behav. Manag. 16, 1–18. doi: 10.2147/PRBM.S382572, PMID: 36636290 PMC9830420

[ref95] Pyne S. C. R. (2017). Emotional intelligence & mental health in the classroom: Experiences of Canadian teachers [Master’s thesis, the University of Western Ontario (Canada)].

[ref96] Qu Y. Yan Z. Chen K. Zhou L. (2024). Exploring the relationship between institutional legitimacy and teachers’ innovative behavior: the serial mediating effects of psychological empowerment and normative commitment. Curr. Psychol. 43, 25377–25388. doi: 10.1007/s12144-024-06237-5, PMID: 40177658

[ref97] Ramirez I. A. L. (2020). Teaching preparedness of pre-service teachers: perception to practice. Int. J. Stud. Educ. Sci. 1, 15–35. doi: 10.46328/ijses.6

[ref98] Regier B. J. (2021). Preservice music teachers’ self-efficacy and concerns before and during student teaching. Int. J. Music. Educ. 39, 340–352. doi: 10.1177/0255761421990787, PMID: 40160997

[ref99] Ryan R. M. (1995). Psychological needs and the facilitation of integrative processes. J. Pers. 63, 397–427. doi: 10.1111/j.1467-6494.1995.tb00501.x, PMID: 7562360

[ref100] Ryan R. M. Deci E. L. (2000). Self-determination theory and the facilitation of intrinsic motivation, social development, and well-being. Am. Psychol. 55, 68–78. doi: 10.1037/0003-066X.55.1.68, PMID: 11392867

[ref101] Salovey P. Mayer J. D. (1990). Emotional intelligence. Imagin. Cogn. Pers. 9, 185–211. doi: 10.2190/DUGG-P24E-52WK-6CDG

[ref102] Sapiee M. L. Abdullah N. A. Halim F. W. Kasim A. C. Ibrahim N. (2024). Exploring the impact of emotional intelligence on employee creativity: the mediating role of spiritual intelligence. J. Chin. Hum. Resour. Manag. 15, 21–37. doi: 10.47297/wspchrmWSP2040-800502.20241503

[ref103] Sawyer R. K. (2011). Structure and improvisation in creative teaching. Cambridge, UK: Cambridge University Press.

[ref104] Sena A. (2020). The influence of organizational culture, job satisfaction, and professional commitment on innovative behavior of flight instructors at the civil flight school in Indonesia. Warta Ardhia 46, 1–17. doi: 10.25104/wa.v46i1.374.1-17

[ref105] Shafait Z. Yuming Z. Sahibzada U. F. (2021). Emotional intelligence and conflict management: an execution of organisational learning, psychological empowerment and innovative work behaviour in Chinese higher education. Middle East J. Manag. 8:1. doi: 10.1504/MEJM.2021.111988

[ref106] Shengyao Y. Xuefen L. Jenatabadi H. S. Samsudin N. Chunchun K. Ishak Z. (2024). Emotional intelligence impact on academic achievement and psychological well-being among university students: the mediating role of positive psychological characteristics. BMC Psychol. 12:389. doi: 10.1186/s40359-024-01886-4, PMID: 38997786 PMC11245800

[ref107] Shu K. (2022). Teachers’ commitment and self-efficacy as predictors of work engagement and well-being. Front. Psychol. 13:850204. doi: 10.3389/fpsyg.2022.850204 35558709 PMC9087840

[ref108] Singh M. Sarkar A. (2012). The relationship between psychological empowerment and innovative behavior: a dimensional analysis with job involvement as mediator. J. Pers. Psychol. 11, 127–137. doi: 10.1027/1866-5888/a000065, PMID: 31409215

[ref109] Sowiyah S. Zulaikha Fitriyanti Z. (2022). The effect of teacher emotional intelligence, teaching facilities and infrastructure on students learning outcomes in inclusive school. United Int. J. Res. Technol. 3, 153–159.

[ref110] Spreitzer G. M. (1995). Psychological empowerment in the workplace: dimensions, measurement, and validation. Acad. Manag. J. 38, 1442–1465. doi: 10.5465/256865, PMID: 23815636

[ref111] Stander M. W. Rothmann S. (2010). Psychological empowerment, job insecurity, and employee engagement. SA J. Ind. Psychol. 36, 1–8. doi: 10.4102/sajip.v36i1.849

[ref112] Su H. Zhang J. Xie M. Zhao M. (2022). The relationship between teachers’ emotional intelligence and teaching for creativity: the mediating role of working engagement. Front. Psychol. 13. doi: 10.3389/fpsyg.2022.1014905, PMID: 36619066 PMC9813491

[ref113] Sultana R. Aldehayyat J. (2018). Career commitment: a mediating link between emotional intelligence and career success. Int. J. Engineer. Technol. 7, 484–490.

[ref114] Sun Q. Yuan Q. (2024). A latent profile analysis of EFL teachers’ self-efficacy: associations with their emotional intelligence and teaching innovation behavior. Int. J. Appl. Linguist. Advance online publication. doi: 10.1111/ijal.12685

[ref115] Sun B. Zhu F. Lin S. Sun J. Wu Y. Xiao W. (2022). How is professional identity associated with teacher career satisfaction? A cross-sectional design to test the multiple mediating roles of psychological empowerment and work engagement. Int. J. Environ. Res. Public Health 19:9009. doi: 10.3390/ijerph19159009, PMID: 35897383 PMC9332691

[ref116] Tabachnick B. G. Fidell L. S. (2013). Using multivariate statistics. (6th Ed.). Boston, MA: Pearson.

[ref117] Thurlings M. Evers A. T. Vermeulen M. (2015). Toward a model of explaining teachers’ innovative behavior: a literature review. Rev. Educ. Res. 85, 430–471. doi: 10.3102/0034654314557949

[ref118] Tripon C. (2023). Navigating the STEM jungle of professionals: unlocking critical competencies through emotional intelligence. J. Educ. Sci. Psychol. 13, 34–47. doi: 10.51865/JESP.2023.1.05

[ref119] Van Den Broeck A. Vansteenkiste M. De Witte H. Lens W. (2008). Explaining the relationships between job characteristics, burnout, and engagement: the role of basic psychological need satisfaction. Work Stress 22, 277–294. doi: 10.1080/02678370802393672

[ref120] Vermeulen M. Kreijns K. Evers A. T. (2022). Transformational leadership, leader–member exchange, and school learning climate: impact on teachers’ innovative behaviour in the Netherlands. Educ. Manag. Admin. Leader. 50, 491–510. doi: 10.1177/1741143220932582

[ref121] Vrontis D. Leonidou E. Christofi M. Hans R. K. Kitchen P. J. (2020). Intercultural service encounters: a systematic review and a conceptual framework on trust development. EuroMed J. Bus. 16, 306–323. doi: 10.1108/EMJB-03-2019-0044, PMID: 35579975

[ref122] Wahyuni W. Sutanto B. Supadi S. (2021). The mediating role of organizational learning in the relationship between organizational commitment and lecturer innovative behavior. JRTI 6, 1–8. doi: 10.29210/3003673000

[ref123] Wajdi M. B. N. Rahayu S. Ulfatin N. Wiyono B. B. Imron A. (2018). The professional competency teachers mediate the influence of teacher innovation and emotional intelligence on school security. J. Soc. Stud. Educ. Res. 9:2.

[ref124] Wang P. Hou Y. (2023). How does commitment affect employee’s innovative behavior? A time-lagged study. SAGE Open 13. doi: 10.1177/21582440231216568, PMID: 40160997

[ref125] Westman M. Hobfoll S. E. Chen S. Davidson O. B. Laski S. (2004). Organizational stress through the lens of conservation of resources (COR) theory. Research in occupational stress and well-being. eds. Perrewé P. L. Ganster D. C. (Bingley, UK: Emerald Group Publishing), 4, 167–220. doi: 10.1016/S1479-3555(04)04005-3

[ref126] Wilson P. M. Longley K. Muon S. Rodgers W. M. Murray T. C. (2006). Examining the contributions of perceived psychological need satisfaction to well-being in exercise. J. Appl. Biobehav. Res. 11, 243–264. doi: 10.1111/j.1751-9861.2007.00008.x, PMID: 40175095

[ref127] Winei A. A. D. Jumrio E. Amianu E. Anggraini Y. (2023). The relationship between psychological empowerment and career commitment of Catholic religious education teachers post the COVID-19 pandemic. JHSS 7, 912–916. doi: 10.33751/jhss.v7i3.9451

[ref128] Wong C.-S. Law K. S. (2002). Wong and Law emotional intelligence scale. Leader. Quart. 13, 243–274. doi: 10.1037/t07398-000

[ref129] Wong C.-S. Law K. S. (2017). The effects of leader and follower emotional intelligence on performance and attitude: an exploratory study. Leadership perspectives. ed. Hooper A. (Abingdon, UK: Routledge), 97–128. doi: 10.4324/9781315250601-10

[ref130] Wright T. A. Hobfoll S. E. (2004). Commitment, psychological well-being and job performance: an examination of conservation of resources (COR) theory and job burnout. J. Bus. Manag. 9, 389–406. doi: 10.1504/JBM.2004.141118

[ref131] Xiaowei W. Juan L. (2019). The impact of psychological empowerment and organizational commitment on innovation performance-based university data analysis. 2019 International Conference on Advanced Education, Service and Management, 3, 527–531.

[ref132] Yang Y. (2023). Challenges in teachers’ professional identity development under the national teacher training programme: an exploratory study of seven major cities in mainland China. Music. Educ. Res. 25, 468–484. doi: 10.1080/14613808.2023.2246136, PMID: 40101104

[ref133] Yao H. Ma L. (2024). Improving teacher career satisfaction through distributed leadership in China: the parallel mediation of teacher empowerment and organizational commitment. Int. J. Educ. Dev. 104:102960. doi: 10.1016/j.ijedudev.2023.102960

[ref134] Yao J.-H. Xiang X.-T. Shen L. (2024). The impact of teachers’ organizational silence on job performance: a serial mediation effect of psychological empowerment and organizational affective commitment. Asia Pacific J. Educ. 44, 355–373. doi: 10.1080/02188791.2022.2031869, PMID: 40101104

[ref135] Yildiz B. Uzun S. Coskun S. (2017). Drivers of innovative behaviors: the moderator roles of perceived organizational support and psychological empowerment. Int. J. Organ. Leadersh. 6, 341–360. doi: 10.33844/ijol.2017.60255

[ref136] Yin H. Huang S. Chen G. (2019). The relationships between teachers’ emotional labor and their burnout and satisfaction: a meta-analytic review. Educ. Res. Rev. 28:100283. doi: 10.1016/j.edurev.2019.100283

[ref137] Yorulmaz Y. İ. Çolak İ. Sağlam A. Ç. (2018). The relationship between teachers’ structural and psychological empowerment and their autonomy. Eğitim Bilimleri Araştırmaları Dergisi 8, 81–96. doi: 10.22521/jesr.2018.82.3

[ref138] Zhang X. Bartol K. M. (2010). Linking empowering leadership and employee creativity: the influence of psychological empowerment, intrinsic motivation, and creative process engagement. Acad. Manag. J. 53, 107–128. doi: 10.5465/amj.2010.48037118

[ref139] Zhang X. Duan X. Wang W. Qin J. Wang H. (2024). The relationship between organizational climate and teaching innovation among preschool teachers: the mediating effect of teaching efficacy. Behav. Sci. 14:516. doi: 10.3390/bs14070516 39062339 PMC11274080

[ref140] Zhang Y. Liang R. Ma H. (2012). Teaching innovation in computer network course for undergraduate students with packet tracer. IERI Procedia 2, 504–510. doi: 10.1016/j.ieri.2012.06.124, PMID: 40177572

[ref141] Zhao H. Zhang X. (2017). The influence of field teaching practice on pre-service teachers’ professional identity: a mixed methods study. Front. Psychol. 8. doi: 10.3389/fpsyg.2017.01264, PMID: 28790956 PMC5522859

[ref142] Zhu J. Yao J. Zhang L. (2019). Linking empowering leadership to innovative behavior in professional learning communities: the role of psychological empowerment and team psychological safety. Asia Pac. Educ. Rev. 20, 657–671. doi: 10.1007/s12564-019-09584-2

